# Some years you live like a coyote: Gendered practices of cultural resilience in working rangeland landscapes

**DOI:** 10.1007/s13280-016-0835-0

**Published:** 2016-11-22

**Authors:** Hailey Wilmer, María E. Fernández-Giménez

**Affiliations:** Colorado State University, Campus Mail 1472, Fort Collins, CO 59043-1472 USA

**Keywords:** Cultural resilience, Life-history, Ranching, Rangeland management, Women

## Abstract

Rangeland researchers are increasingly interested in understanding working rangelands as integrated social–ecological systems and in investigating the contexts of human decision-making processes that support system resilience. U.S. public lands ranchers are key partners in rangeland conservation, but the role of women in building system resilience has not yet been explored. We conducted life-history interviews with 19 ranching women in the Southwestern United States. We analyzed the resulting transcripts by identifying contradictions between women’s material practices and traditional discourses in the ranching livelihood that illustrated women’s efforts to maintain both a way of life and a living during social and ecological change. These gendered practices of cultural resilience included self-sacrifice during difficult financial times, engagement with non-rancher networks, and efforts to transfer cultural and technical knowledge. We argue that the key part ranchers play in rangeland conservation cannot be fully understood without a consideration of gendered practices of cultural resilience.

## Introduction

Changes in Southwestern U.S. rangeland landscapes challenge the ranching communities and families that rely on these systems for their livelihoods. Made up of grasslands, shrublands, and savannahs with grazing potential and managed as natural ecosystems (Society for Range Management 1998), rangelands are working landscapes (Huntsinger and Oviedo 2014), which support ranching-based cultures, communities, and livelihoods as well as biodiversity.

Recent research holds that well-managed extensive rangeland-based livestock production systems, working rangeland landscapes, can provide high-quality protein for human consumption while maintaining connected, diverse landscapes (Plieninger et al. 2012; Charnley et al. 2014; Huntsinger and Oviedo 2014; Roche et al. 2015). Knight (2007) has argued that ranchers are a “keystone species” in Western U.S. conservation because of their roles in maintaining biodiversity and landscape connectivity (Knight 2007; Brunson and Huntsinger 2008). Barriers to ranch succession and financial viability, conflict among rangeland stakeholders, and the decline of ranching communities are therefore conservation issues, because they threaten the continuity of working rangeland landscapes (Lubell et al. 2013; Charnley et al. 2014; Knapp et al. 2015).

Western U.S. ranching communities that work in rangeland landscapes face extreme weather variability, shifting rural demographics and economic opportunities, volatile commodity prices, and an uncertain regulatory environment (Briggeman et al. 2007; White et al. 2009; MacDonald 2010; Johnson 2011; Pugh 2012). Changing government policies, new technologies, and market competition have increased the size, reduced the number, and altered the structure of American family farms and ranches (Barbieri et al. 2008). However, in 2012, individuals or family operated 80 % of U.S. beef operations (US Census of Agriculture 2015). To understand the processes of rangeland change, researchers turn to an exploration of resilience, the capacity of a system to absorb disturbance and retain its basic structure and function (Walker and Salt 2006).

Here, we focus on social resilience, or “the ability of groups or communities to cope with external stresses and disturbances as a result of social, political and environmental change” (Adger 2000, p. 347; Brown 2014). Social resilience is often examined from an outsider’s perspective without understanding how community insiders view resilience. We investigated gendered cultural resilience as described by women cattle ranchers in 19 life-history interviews conducted in New Mexico and Arizona, USA. Our research objective was to document how ranching women maintained livelihoods that supported both a living and a way of life during social and ecological change (Crane 2010). We document how women ranchers foster cultural and ecological resilience in Southwestern U.S. rangelands via their adaptations to financial instability and uncertain succession planning, and their creation of new social networks.

## Background

### Resilience: Gendered contexts of a concept born in ecology

Social–ecological systems (SES) theory conceptualizes relationships between human and natural systems as an integrated system composed of human and natural dynamics (Berkes et al. 2000). SES theory seeks to understand the source and role of change in complex social–ecological systems and to “live with,” rather than control, complexity through adaptive, experimental management and social learning (Holling and Meffe 1996; Holling and Gunderson 2002).

A key aspect of system complexity, resilience helps us understand how systems recover after a disturbance (Walker and Salt 2006). The term resilience originates in ecology, where it refers to how ecological systems respond to change (Holling and Meffe 1996). But in the increasingly uncertain and complex environment of ranching communities in the Southwestern U.S., social resilience is as important as ecological resilience to the viability of the ranching way of life.

Linking a theory with ecological roots to social experiences is challenging. Social scientists criticize resilience theory for ignoring the context of ecological knowledge and for failing to explore systems of power while emphasizing institutional design and rule-making (Brown 2014; Olsson et al. 2015). Cote and Nightingale (2012) question the effectiveness of analyzing social resilience by simply documenting local or indigenous knowledge. They advocate for placing knowledge in social and cultural context, and exploring the multidimensional social processes, relationships, and identities that influence decision-making in these systems (Cote and Nightingale 2012).

Gender is one category of social identity through which rangeland scholars can explore the socio-cultural context of rangeland system change. A dynamic, complex, and social performance that intersects with other experiences, gender distinguishes men and women into social categories but does not dictate group membership (Young 1994; McCall 2005; O’Shaughnessy and Krogman 2011). Gender is an under-examined, complex, and deeply personal experience with implications for broader social power asymmetries. Thus, it provides an important starting point in the effort to contextualize the social processes driving change on rangeland systems.

Research on gender in both the rangeland literature and social–ecological system resilience is nearly absent in the context of the Western U.S. Much of the work exploring women in natural resource systems has been focused on developing nations (Coppock et al. 2011), Australia (Farmar-Bowers 2010), or farming systems in the United States (Trauger 2004; Barbercheck et al. 2014). Farmers and ranchers have been traditionally distinguished on cultural and productive terms in the Western United States. Gender in the livestock industry (Pilgeram 2007), and within extension or agriculture education programs (Trauger et al. 2010; Enns and Martin 2015), has received some attention. However, there is mounting evidence that women drive change in rangeland systems (Coppock et al. 2013) and that global climate change impacts are gendered (Nelson et al. 2002; Alston 2010). However, to our knowledge, no one has examined gendered resilience practices in working rangeland landscapes of the Southwestern U.S.

### Theoretical framework

To address gaps in current research, we draw from three main areas of the literature. To explore resilience as an embodied practice, we adopt the concept of cultural resilience developed by Crane (2010). To identify examples of gendered cultural resilience, we use O’Shaughnessy and Krogman’s (2011) analytical framework, which identifies contradictions in women’s lived experiences of change in natural resource-based communities. We also use a narrative analysis methodology rooted in feminist theory (Squire 2013).

Crane’s (2010) concept of cultural resilience refers to how individuals maintain livelihoods that support both material and moral needs in the face of multiple stresses and shocks. An emic approach, or an analysis of cultural phenomena from the perspective of someone inside the culture being studied, allows Crane to analyze socially constructed meanings and normative values around resilience from the perspective of local people. We employ Crane’s emic approach, apply his assumption that peoples’ way of life has meaning to them, and view resilience as a matter of sustaining livelihoods that support both material and cultural needs.

Next, we adopted a framework which draws extensively from feminist literature. The framework considers gendered cultural practices, with the premise that gender is material, discursive, and often contradictory. Under this view, gender includes practices related to social structures, conditions, and relationships that shape daily life and the physical environment (material practices), and practices that shape the production and reproduction of ideologies, stereotypes, and cultural norms (discursive practices) (O’Shaughnessy and Krogman 2011). This emphasis on contradictions stems from a shift in gender research toward analyzing gendered practices that reveal the everyday meaning of women’s lives and avoid universalizing women’s experiences (McCall 2005; O’Shaughnessy and Krogman 2011). The authors note that material-discursive contradictions help scholars examine the relationship between practices and conditions as they relate to beliefs at the community level. In using this framework, we recognize the complex intersection of culture, livelihoods, and gender. We also acknowledge that gendered experiences may contradict cultural perceptions of gender in the Western U.S. rangeland context, where women have been categorized as strong, independent “career women,” but face both physical and social inequalities, including gendered barriers to access to credit and inheritance of ranch lands (Wilmer and Fernández-Giménez 2016).

We chose a narrative methodology and life-history interviews to gather and present women’s voices (Daly 2007). The methodology reverses the conventional researcher–subject power dynamic and invites greater participant agency in the research (Lieblich et al. 1998; Squire 2013). It allows the researcher to recognize that the data from interviews are an interpretation of women’s experiences and that many interpretations of the same experience may exist (Daly 2007).

## Materials and methods

### Recruitment and data collection

We recruited self-identified ranch women who grazed cattle on public lands ranches in Arizona and New Mexico, U.S. To identify study participants, we relied on community gatekeepers and then asked participants to refer us to other women in their networks. Those women who were not retired were all public lands ranchers and had varying levels of dependence upon public grazing permits and the forage these lands provide (Tanaka et al. 2005). Grazing by permit on public lands continues in a highly contentious political environment in the Western U.S. Various interest groups have pressured for the elimination or reduction of grazing permits because of concerns for the ecological and social impacts of grazing and ranching practices, particularly related to endangered species and profitability (Fleischner 1994; Pugh 2012). Advocates of public lands grazing and government agencies that administer grazing permits cite economic, socio-cultural, and ecological benefits of public lands grazing (Bradford et al. 2002; Pugh 2012).

The first author conducted and audio-recorded interviews with 19 ranching women, aged from 28 to 85, in the summer of 2013 under approval of Colorado State University’s Internal Review Board (IRB) for human subjects research (protocols 10–1829H, 11–3178H and 12-3381H). To prompt the narrative, she asked participants to tell their life stories. She asked them to cover early life, family and ranch history, ranching practices, changes on the ranch, and views of the future. Interviews were transcribed verbatim and checked against the audio records for accuracy, and we replaced names in the transcripts with pseudonyms. The first author also conducted six weeks of participant observation on seven ranches in 2012 and 2013.

### Data analysis

Our narrative analysis took an experience-centered approach (Squire 2013) to locate gendered practices of resilience in the interviews. We first identified complete stories in each interview, or coherent narratives separated by a change of subject, character, or timeline. Using a spreadsheet, we coded each story to mark (a) a main topic and (b) contradictions between ranching discourses and women’s material practices in the story. We identified patterns by checking our initial codes from single stories against the context of each whole interview transcript (Lieblich et al. 1998). We sorted the contradictions into thematic groups and selected the three most dominant themes. To ensure validity during this process, we engaged in prolonged immersion in the data, triangulation with participant observation notes, negative case analysis, peer debriefing, reflexive writing, and member checking (Lincoln and Guba 1986). Transcripts and results were mailed to participants for their review.

## Results

Our analysis revealed three resilience practices (Table 1). Each is an example of gendered cultural resilience identified by a contradiction between discourses in ranching culture (discursive practice) and women’s material practices. Below, we briefly describe and illustrate each practice with supporting data from our interviews.

**Table 1:** Each gendered cultural resilience practice demonstrates women’s resilience to a change in the system through a contradiction between traditional ranching discourses and women’s material practices

	Practice 1	Practice 2	Practice 3
Traditional ranching discourse	Ranching is an important livelihood and an identity for ranching families	Ranchers are fiercely independent and self-sufficient	Ranching is facing a succession crisis
Driver of change in ranching systems	Uncertain climate, livestock health, and market conditions create financial instability for ranching families	Increased regulation and conflict on public lands ranches require ranchers to engage with non-ranchers and the political process	Social and ecological uncertainties (including tax, climate, and land value change) make it difficult for young people to go into ranching
Women’s cultural resilience practice	Women lessen their own standard of living for ranch ecological and/or economic sustainability	Women bridge ranching and non-ranching worlds through advocacy and community keeping	Women produce and reproduce ranching knowledge; empower younger generations to chose to stay in the ranching

### Resilience practice 1: Some years you live like a coyote

“Women persevere. You know, the women do. I mean the women are the ones that figure out 14 ways to cook beans and 19 different ways to serve hamburger, because you got to have a trailer or you get one pay check a year, or two, we do two on our operations. But you know I remember when [my husband] and I got married and I was telling him what he was getting into because I knew and he didn’t. You know he had a little more of a romantic, he doesn’t have it now. [Laughing] But he did have more of a romantic view of what ranching was going to be. And [my husband] told me, he said ‘I refuse to live like a coyote.’ And I said, ‘No. When you ranch there are some years you live like a coyote.’

And this last year, I don’t know, he was kind of emotional, [my husband’s] not an emotional man, and he said, ‘I don’t think that we can make it.’ And I said, ‘We can. We’re going to live like coyotes.’ We are in our third process of cutting our expenses in half. You know the days of having new pick-ups, we never did do a new pick-up every year but we did about ever 3 or 4 years. Those are over. But my husband has changed, in that it’s more important to him now to have the ranch than it is to not live like a coyote, but mainly because we have a granddaughter who has what I call the dirt in her blood.” (Wendy, New Mexico)

Throughout the interview we conducted with Wendy, she described her ranching livelihood as a vital way of life, a core part of her identity. It was “in her blood.” When she recorded this narrative in June of 2013, New Mexico was desperate for rain. In the clutches of the hottest drought on record, Wendy described how ranching women would help their families persevere to the next monsoon season. As she noted, cow-calf ranching households may budget around a single influx of income each year when the calf-crop is sold. The women in this study also identified the drivers of financial difficulty in their households to include family health, inter- and intra-annual variability in temperature, and the timing and amount of precipitation, as well as livestock health and nutritional problems.

All the women in the study chose to take less material benefit for themselves in terms of profit, standard of living, or nutrition, to support some aspect of ranch sustainability, including the condition of livestock, rangeland, and/or ranch infrastructure. Five women described working or attending job training off-ranch to fill gaps in ranch income or health insurance for their families. Even while spending most of their day off-ranch, these women maintained identities as ranchers and as active participants in production agriculture.

In response to difficult financial times, women also reimagined the overall structure of their ranching operations, sometimes engaging with different paradigms of rangeland management and livestock production, or downsizing. Some adopted goals to improve multiple, interconnected processes within rangeland ecosystems rather than focusing only on production and financial goals. Two of the ranchers developed goals and monitoring approaches for framing and adapting their management holistically. Other ranchers reorganized their relationships to markets by implementing direct-marketing or diversifying livestock income by raising, training, and selling horses.

### Resilience practice 2: Staying independent by staying connected

“We have so many meetings we have to go to, you can’t be like some people and just put your head in a hole in the sand and ignore these problems and let someone else take care of it for you all the time. We’re the people who have devoted our life, many, many years, I mean our ranch infrastructure has really suffered because we’ve put so much time in with our local conservation districts, and the University, and I call it community service, it’s our way of community service. It’s important for our operation too.” (Edith, Arizona)

The second theme highlights the tension between a discourse of self-sufficiency in ranching communities and women’s need to connect to broader social networks. Today, complex cultural and political contexts in the Southwest U.S. are continually reshaping rural landscapes, land management policy, and livestock markets. A common narrative running through ranching communities is that ranchers should be independent, self-sufficient, and rely on their own labor, resources, and skills rather than help from government agencies. However, recent increases in regulation, public attention to ranch management, recreation, and other competing uses of public land threaten ranchers’ independence and autonomy.

While ranching discourse reflects a culture of fierce independence threatened by outsider influence, the women ranchers we interviewed recognized the gains in social and political capital they make by linking with non-rancher networks. Across the interviews, women described outreach activities and leadership roles with educational, scientific, political, and community service organizations, as well as efforts to educate non-ranchers about their industry and operations. Some identified as activists, working to change policy and public opinion about ranching. Others worked to build collaborative networks among diverse stakeholders that would support ecosystem management of their public lands ranches or help build allies. Seven women described this tension between independence and connection as a complex issue in their lives, but all the women we interviewed invested time in community keeping, outreach, or advocacy.

For example, Edith was highly involved in livestock industry advocacy, and supported her husband’s successful career in wildlife biology. She also emphasized the value of self-sufficiency to her identity and ranch profitability. She and her husband performed all ranch labor themselves, lived off the power grid, and declined to participate in government grant programs. Edith argued that government grants would reduce their autonomy. She reconciled the tension between independence and connectivity by citing the benefits of involvement to her children and ranch. This involvement was so important to her that she expressed disgust at other ranchers who do not take the time to be involved in activities that benefit the ranching community as a whole.

### Resilience practice 3: Building a future on foundations of knowledge

“The goal for our family is that this ranch can be free of debt and operate with some sort of a system that [our children] can kind of stay on that system. We have all of our kids out when we brand. They’re all good hands and we all drag ‘em to the fire and flank and every one of ‘em can do that pretty well, girls and boys. We got a little granddaughter that gets right in the middle of it. That’s a thing that we like, that our family, all of our kids, have learned. They all do different things now, but I think if they chose to do this I think they’ve got enough of a background that they can learn like I did as I went along just because I had the right foundation in ranching.” (Laura, New Mexico)

During field work, we heard a discourse in ranching communities that a crisis exists in ranch succession. High ranch land values (created in part by amenity and development buyers), weather, tax and regulatory uncertainties, the challenges of rural life (including barriers to healthcare access and education), and ranching’s dangerous physical work environment were all identified as barriers to passing on the ranching way of life to the next generation. This concern was explicitly identified and reconciled in the narratives of seven women but discussed in all 19 interviews.

**Fig. 1:**
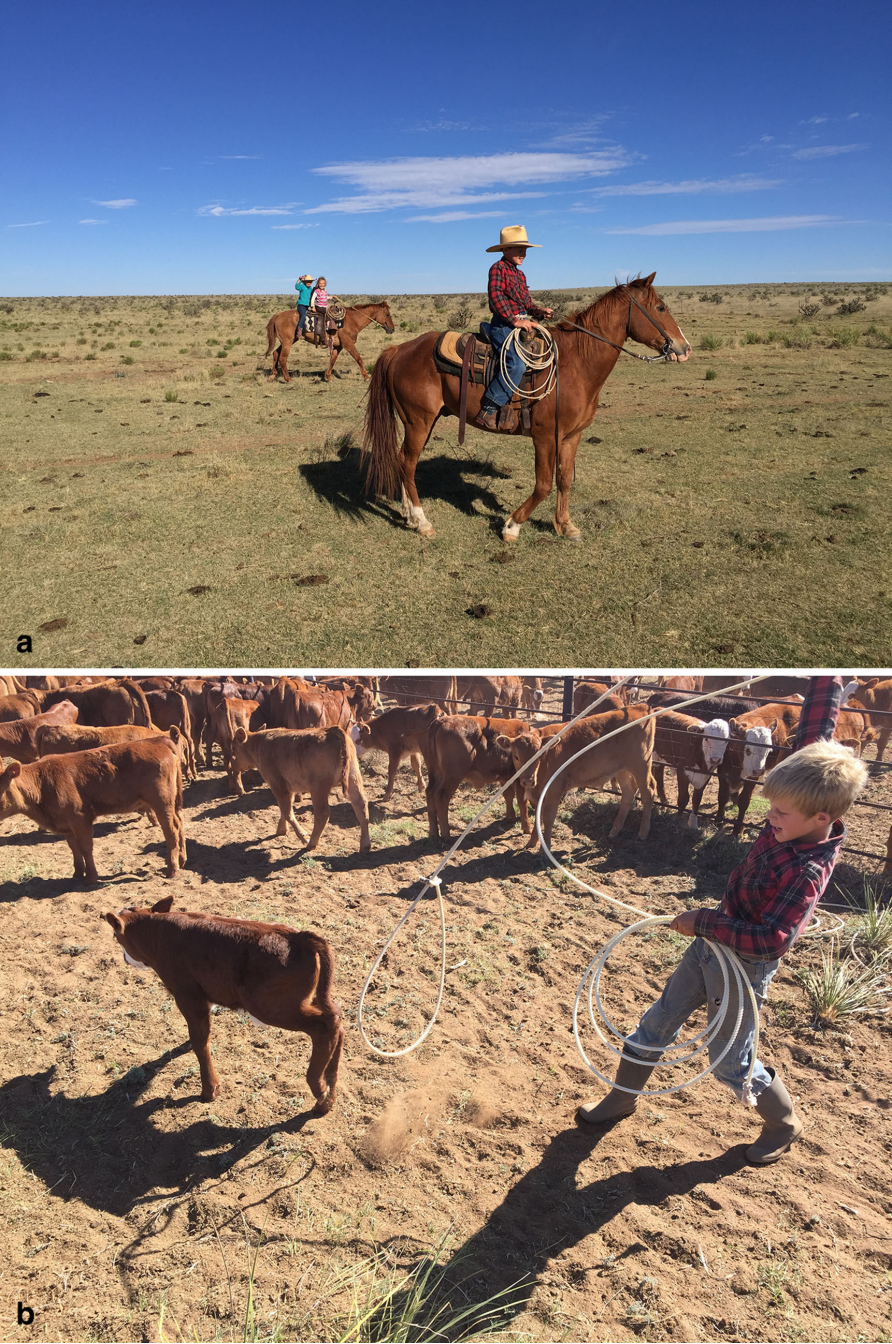
**a** Children in New Mexico, U.S. ranch gain skills in livestock handling and horsemanship with mentoring from mothers and grandmothers; **b** A young boy learns to rope cattle on New Mexico, U.S. ranch (Photos by Pat King)

Women’s material practices contradicted the discourse that ranching faces a succession crisis, through direct efforts to produce and reproduce ranching cultural knowledge and empower younger generations to choose to stay in ranching. In her quote above, Laura referred to teaching her grandchildren traditional branding practices (Fig. 1a, b). She said that if they learned these skills, they would have a foundation to come back and operate the ranch in the future. Another woman, Sandra, described the cultural knowledge that ranching children learn early in life:

“It involves the fact that ranching is not a job. It’s a culture. Some urban kid cannot say, hey, I’d like to be a rancher. It’s absorbed, how you move those cows, how do you know where to move when the gate’s open and the herd is there, it’s almost a sixth sense, and an instinct. Our kids learned more than our grandchildren have learned. Will our grandkids learn it? They won’t learn it from their parents, their parents are in Los Angeles.” (Sandra Arizona)

**Fig. 2:**
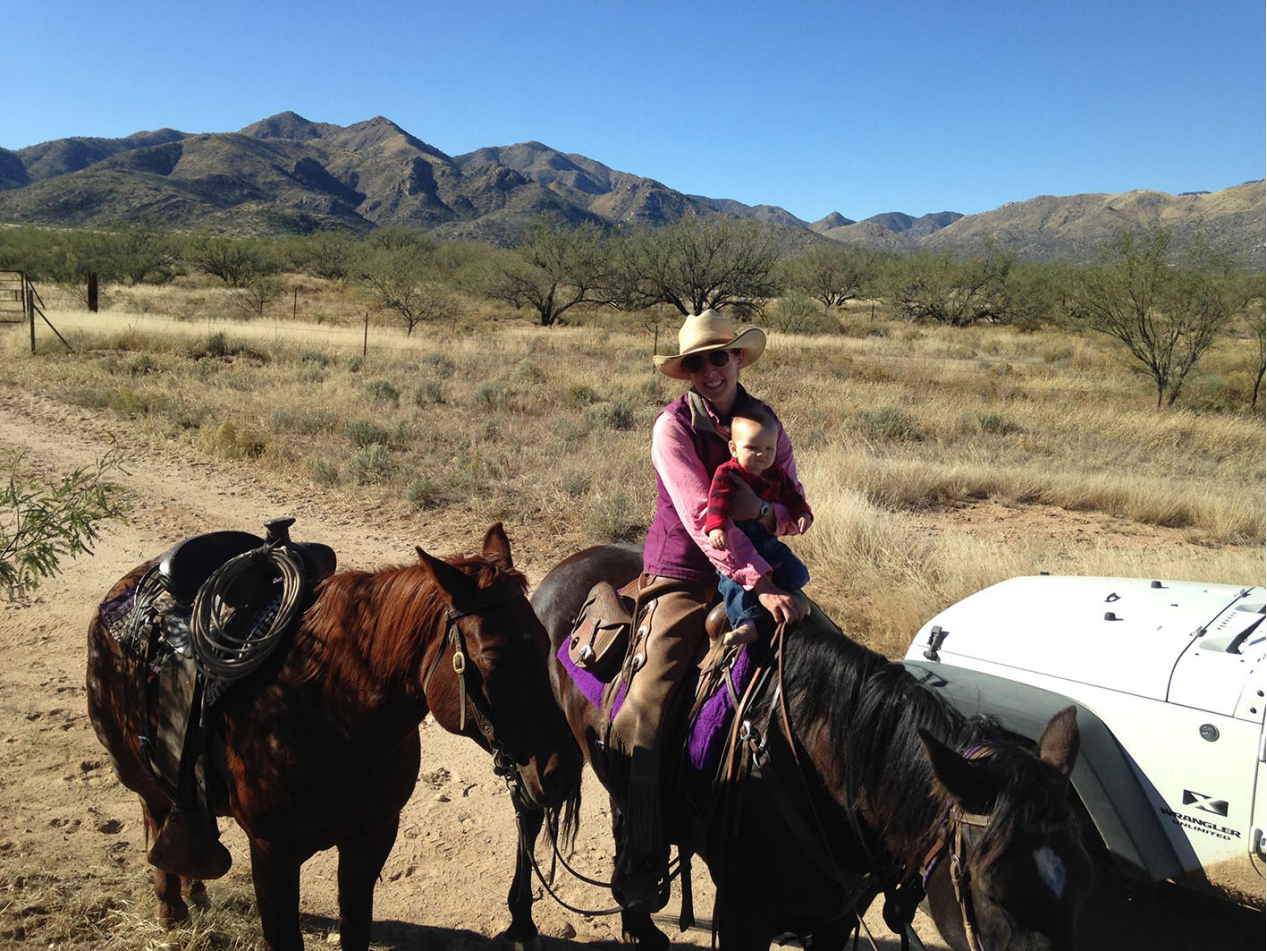
Arizona, U.S. ranch woman with young child participates in cattle management activities (Photo by Sarah King)

Throughout her interview, Sandra discussed her role in helping younger generations stay connected to ranching (Fig. 2). But she maintained that her children should be able to choose to come back to the ranch and not be pressured to return. As with Sandra and Laura, the interviews revealed resistance to a seemingly unanswered question: what is the future of the family ranching way of life? Ranching women practiced cultural resilience by empowering youth with cultural knowledge and freedom of choice.

## Discussion

Using Crane’s (2010) cultural resilience concept, a narrative methodology and feminist analytical framework, we documented gendered practices of cultural resilience. These included women lessening their own standard of living to maintain ranch financial viability, women bridging ranching and non-ranching communities through outreach and advocacy roles, and women addressing ranch succession through mentoring and guidance of younger generations. This study supports the need to gather voices of diverse rangeland stakeholders through methodologies that help researchers build partnerships with land managers (Sayre 2004). This includes the need to seek perspectives from individual members of ranch families rather than studying only male heads-of-households (Fulton and Vanclay 2011).

Our results provide insights into the under-examined roles of women in these ranching systems. It is well known that ranchers face an opportunity cost to go into ranching, and scholars have documented the non-economic motivations of ranchers, including lifestyle and heritage (Smith and Martin 1972; Tanaka et al. 2005). But little is understood about the meaning of choosing a ranching lifestyle that is financially unstable. The life-history narratives gathered in this study show how women took on responsibility for cultural resilience in specific, gendered ways. While resilience practices were not exclusive to women, the interviews revealed cultural norms that women, rather than men, keep and transfer knowledge of these practices, in part because of women’s attention to the long-term financial viability and ecological sustainability of their ranches.

Narratives addressing independence and connection illustrate how women practice resilience by negotiating conflicting cultural and political needs. By serving as community leaders, women took agency in the face of social change, but this work to stay connected was done alongside, or even to support, a sense of self-sufficiency. The women who resolved this issue in their narratives cited the importance of community engagement to the viability of their ranching way of life. Our results suggest that ranching discourses may be changing in some communities and families around isolation and self-sufficiency, and that isolation from the broader community or non-ranchers may be becoming less appropriate. Women’s industry groups have long been important to social and political experiences of ranching women in the Southwest U.S., but women’s political consciousness merits further study specifically examining how women’s industry groups or advocacy roles shape resource access, control, and management of U.S. rangelands (Alston 2010; Rocheleau et al. 2013).

Our approach has a number of limitations, including a small sample size, less generalizability than quantitative studies, and a sample potentially biased toward ranch women with an interest in research or advocacy. Our study did not consider race, ethnicity, sexuality, or ability, intersecting identities (McCall 2005) that frame resilience practices on rangelands and merit further research. However, the study does present an analysis of diverse perspectives that can challenge future social–ecological research to consider the role of gendered cultural resilience practices in social–ecological system resilience. Previous studies have documented the decision-making roles and experiences of farm women (Trauger 2004; Farmar-Bowers 2010; Alston 2014). Our study contributes to this body of literature by documenting the specific practices of women that help to maintain viable extensive beef production systems that operate with public lands grazing leases in the Western U.S. In these systems, critical areas of biodiversity and landscape connectivity are maintained on working ranches though public and private partnerships (Charnley et al. 2014). This study documents the role of women in maintaining resilience in these systems.

### Implications for resilience theory

Does analyzing resilience on a gendered, cultural level contribute to our understanding of resilience at the whole-system scale? We argue not only that it can, but that the consideration of resilience at this scale is an important missing link in SES scholarship. While SES scholars have examined cognitive, institutional, and broader social decision-making processes, the gendered and social context is under-explored. Specifically, a major emphasis in resilience literature has been on the design and function of institutions (Ostrom 1990; Berkes et al. 2000; Bestelmeyer and Briske 2012). Decision-making studies in rangeland and agricultural science focus largely on innovation adoption by identifying demographic predictors of rancher innovation adoption decisions (Coppock and Birkenfeld 1999; Rogers 2010). Both the institutional and innovation adoption approaches have a limited capacity to explain decision-making patterns of individual land managers and can be enhanced by qualitative methods that explore the multiple contexts and experiences of decision-makers (Sayre 2004).

### Implications for rangeland landscapes

Rangeland research that examines the role of ranchers in biodiversity and ecosystem conservation may help us understand how gendered cultural resilience practices can shape the function and structure of rangeland landscapes. Intact, connected, and extensive rangeland ecosystems support a number of ecosystem services, including open space; wildlife habitat and soil, air, and water quality; a sense of place; and cultural heritage (Bestelmeyer and Briske 2012; Sayre et al. 2013; Huntsinger and Oviedo 2014). Rangelands are most threatened by conversion to cropland, residential, or industrialized uses (Brunson and Huntsinger 2008; Sayre et al. 2013; Sylvester et al. 2013), and by management practices such as grazing intensification and fire suppression that alter species composition and homogenize complex, patchy landscapes (Fuhlendorf et al. 2012).

While this study did not include an analysis of biophysical data, the interview data present women’s perceptions of the impact of their practices on rangeland management. Women’s practice of lessening their own standard of living during difficult financial periods potentially reduces demands on rangeland forage resources in the short term. The women also discussed their roles in reorganizing rangeland management on their ranches as a response to these lean times. Women’s efforts to bridge ranching and non-ranching worlds through advocacy and community keeping maintain social networks that link ranchers to new information, adaptation strategies, and social resources. These resources can help ranchers adapt to or cope with shocks to ranching operations, such as drought, and can support the mid-to-long-term financial viability of extensive rangeland use. Finally, women’s facilitation of ranch succession potentially helps to perpetuate family ranching land uses and stewardship practices at a particular scale, buffering diverse private lands from development or consolidation into larger ranches. The key roles ranchers play in conservation cannot be understood without a consideration of gendered practices such as those we have identified in this study.

## Conclusion

In conclusion, we have identified three gendered practices of cultural resilience that women ranchers use to adapt to change in ranching systems of the Southwestern U.S. Each practice is identified by a contradiction between discourses in ranching culture (discursive practice) and women’s material practices. First, to adapt to uncertain financial situations in ranching, women lessen their own standard of living for ranch ecological and economic sustainability. Second, as increased regulation and conflict on public lands ranches require ranchers to engage with non-ranchers and the political process, women bridge ranching and non-ranching worlds through advocacy and community keeping. Finally, as ranching faces a potential succession crisis, women produce and reproduce ranching knowledge and empower young people to choose to go into ranching. These practices are social and ecological contributions to resilience. By examining the complex and contradictory practices of decision-makers in rangeland SES systems, resilience scholars can better understand the social processes that shape how institutional rules are applied because managers’ adaptive action is situated within gendered contexts.
